# Weighing benefits and risks in aspects of security, privacy and adoption of technology in a value-based healthcare system

**DOI:** 10.1186/s12911-018-0700-0

**Published:** 2018-11-13

**Authors:** Edward Meinert, Abrar Alturkistani, David Brindley, Peter Knight, Glenn Wells, Nick de Pennington

**Affiliations:** 1 Healthcare Translation Research Group, Department of Paediatrics, University of Oxford, Oxford, OX3 9DU UK; 2 Department of Infectious Disease Epidemiology, Faculty of Medicine, School of Public Health, Imperial College London, London, W6 8RP UK; 3 Oxford University Hospitals NHS Foundation Trust, John Radcliffe Hospital, Headley Way, Headington, Oxford, OX3 9DU UK; 4 Oxford Academic Health Science Centre, Oxford, OX4 4GA UK; 5 Global Digital Health Unit, Department of Primary Care and Public Health, Imperial College London, London, W6 8RP UK

**Keywords:** Delivery of health care, Value-based healthcare, Technology adoption

## Abstract

**Background:**

Technology can potentially enable the implementation of a value-based healthcare system, where the impact of quality of care is offered at optimised cost for maximised patient benefit. Technology can deliver value by aiding in data collection to evaluate outcomes and measure costs on a patient and population level. Healthcare organisations, however, face several challenges and risks that result almost exclusively from the use of these technologies.

**Discussion:**

Some challenges associated with healthcare technology include their unsustainability, due to lack of scale-up plans and timely evaluations. Other risks include noncompliance with data protection policies, inadequate data governance, and overestimated expectations resulting from the rapid introduction of new technologies.

**Conclusion:**

Organisations need to consider the risks and challenges associated with the use of technology and develop comprehensive strategies that mitigate factors leading to non-adoption and to realise benefits for achieving a value-based healthcare system.

## Background

Value, in a value-based healthcare system, refers to the delivery of the best quality care in the most cost-efficient way [1]. Technology, defined as the use of information systems for the administrative management and delivery of care, is one of the three enablers of a value-based system [1], but there remains a significant gap between the current use of technologies for this purpose and the potential they could offer [2]. Health technologies are developing at a rapid pace and while it is possible to envisage ways in which they can impact cost-effectiveness (in comparison to legacy paper-driven approaches and other more manual methods), they also subject systems to new risks and challenges. The way these issues impact a perceived notion of value requires further consideration. Questions also remain regarding the sustainability of healthcare technologies. Several sources agree that often the key challenge with health technologies is not in the design or the innovation itself, but in the lack of policies and frameworks that can enable adoption, sustainability and scalability [2–4]. Security and privacy-related challenges also remain one of the most significant concerns for creating a technology-led value-based healthcare system. An increase in data collection and sharing creates patient privacy concerns due to the potential of unintended use – digital systems can be subject to non-compliance with information governance regulations, data breaches and cyber-attacks. This article will explore some of the risks and challenges associated with the use of technology to consider what impact they could have on the measurement of value. Perspectives on the way in which these impact management and planning in value-based healthcare are derived from the authors experiences in the delivery and management of healthcare IT.

## Discussion

As healthcare systems leverage technologies to create a value-based system, assessment and evaluation of such technologies are paramount for their continuation and improvement. However, evaluation of healthcare technology is not straightforward, as it requires examination of factors including engineering, strategic implementation, uptake and cost. Suppose we consider a fundamental aspect of value centred on the economic benefit derived from more efficient systems and broad adoption. While on one hand it may seem straight forward to capture this through implementation of a quantitative model, on the other hand there are many other variables which impact medium to long-term sustainability and use. These dimensions involve the use of agile implementation approaches that do not necessarily lend themselves to modelling which is static or predictable. For example, technology “scale-up” plans need to be implemented in the design process and technologies need to be tailored to users’ needs to ensure sustainability [4]. The ability to measure such plans, either in time to implement, resources required, quality and other factors is subjective and highly variable depending on organisational priorities and needs. Additionally, it is argued that abandonment of technologies can occur due to the deficiency in creating a “policy framework” alongside the innovation itself to provide effective and safe use of the technology [2] – developing such frameworks are a significant undertaking in their own right, and easily overlooked when emphasis can be centred on the design, development and implementation of a new technology. Since health information technologies evolve and are produced at a fast pace, evaluations and assessments of the technologies may not occur at the same rate of change indicating a need for more rapid assessment [5]. Assumptions change over time; a system installed today is likely to be out of date in a short period later, so in contrast to systems with longer lifecycles, this makes benefit modelling more difficult. For sustainability of technologies in creating a value-based healthcare system, it is suggested to introduce the technology in “manageable increments” while considering long-term arrangements [1]; these approaches are combinations of methods which make its perceived impact on value one which is hard to record consistently. While an information technology professional may see value in reduction of manual processes, a clinician may see a loss of value in loss of direct contact with patients and a policy maker may take different positions dependent on the use case. This variability in perception of what defines, and influences value make the concept difficult to memorialise.

A primary concern in using data to support value-based approaches results from information governance. Both the European Union’s General Data Protection Regulation (GDPR) [6] and the USA’s Health Insurance Portability and Accountability Act (HIPAA) [7] advocate heavy penalties for organisations who fail to store and secure their user’s data appropriately. However, frequently there appears to be a misuse of patient data or non-adherence to policies. For example, in 2017, a National Health Service (NHS) hospital, Royal Free, shared patient data of more than a million patients with an independent data processing organisation, DeepMind Health [8]. The Trust shared patient data without explicit patient consent for this kind of implementation case, in contravention of data protection laws and information commissioner guidance [8]. While it was recognised that both organisations undertook the data sharing in the pursuit of improved patient care, this example demonstrated the risk to confidence from inadequately obtaining patient consent. Even if data sharing is performed for the sake of “public interest”, not achieving explicit patient consent could exasperate concerns about data collection [9]. This is especially true since many patients may not mind the sharing of their data as long as they are properly informed [9]. Assuming such issues concerning access, can be addressed, what impact does this form of data sharing have on value? While the ability to use aggregate population-level data to create insight has clear cost-benefit potential, in what ways does the principle of value translate to the ethical and reputational issues advanced by information governance concerns?

With increased storing and sharing of patient data, maintenance of cybersecurity is imperative. Cyber-attacks are a key challenge that organisations storing patient and hospital data face. Cyber-attacks can happen when entities from outside or inside the system disrupt or interfere with networks for access and are especially concerning if the whole system is impacted; an example of such attacks is through use of malware [10]. With healthcare systems aiming to create value-based systems, increased population-wide patient data from multiple resources are being collected. While patient data from different sources including technologies such as the Internet of Things (IoT) and aggregated data insight collected from sources using Artificial Intelligence (AI) methods have the potential to significantly improve healthcare outcomes [11, 12], they can also subject organisations to become targets for cyber-attacks. In addition, compared to other industries, healthcare systems are generally lagging behind in human-centred countermeasures through effective employee training and prevention of access to malicious external documents/files [13]. An example of a recent cyber-attack was the WannaCry malware incident which affected 80 NHS trusts and more than 600 different National Health Service (NHS) organisations in England [14]. Computer use, patient care and even medical equipment were all hindered by this security vulnerability [15]. Although it was possible to prevent access to patient data from this malware, such attacks raise concerns about the security of electronically recorded data and may affect patients’ and the public’s trust in data sharing and highlight a need for increased vigilance, training and safeguarding against this kind of security threat.

New technology has a particular life cycle that determines its sustainability as described in Gartner’s hype cycle, which suggests that it is likely that a technology can be abandoned even after being linked to significant system-wide benefits [16]. There are many examples of technologies that were developed for use in healthcare settings but were eventually abandoned for different reasons such as non-adoption, unsustainable funding and inability to create scalable technologies [3, 4]. For example, in 2013 IBM Watson, a cognitive computing system which allows clinicians to enter both structured and unstructured data and uses these inputs for problem solving and providing informed-decision making [17] was announced to be used with by a leading cancer research institute, MD Anderson [18]. Previously, the IBM system had gained popularity as a result of winning the trivia game “Jeopardy”, demonstrating its ability to use computational processes to defeat human competitors [19]. The general premise of the use of the system was that such computing processes could be used to augment and extend capabilities in cancer detection and diagnosis; doctors and researchers at the institute used Watson to assist in diagnosis in the personalized treatment of cancer [17]. In less than six years’ time, these plans were put on hold, revealing several faults in the project implementation [18]. While Watson does herald potential to improving health research through its implementation of sophisticated computational process, in this instance it fell short on meeting research needs and being a viable ongoing concern. The key issues included challenges in data integration, engagement, cost of delivery and project scope. [18]. Making sure these kind of projects are sustainable and designed effectively requires much more than technological innovation, but a lifecycle approach to ensure projects meet objectives. Value must be understood in view of benefits but also examining the mechanisms necessary for system enablement. These issues demonstrate the challenges of the rapid adoption of technology and potential unintended consequences.

## Conclusions

To create a health system that is striving to deliver value through the use of technology, there are several challenges and risks that need to be addressed. The impact that these issues has on the definition of value is one which must be considered, otherwise the impact of technology could be overestimated, and benefits reduced due to changing circumstances. Digitising an organisation with the size and complexity of those in the healthcare creates a considerable challenge in setting realistic goals and timelines while managing the adaptive change required of the existing individuals, systems and processes in the organisation. The risks and challenges associated with technologies do not infer the need to set technology aside and try to improve care without it. In contrast, technology immersion in healthcare is inevitable and is needed more than ever. The increased incentives worldwide to adopt healthcare technologies and the fact that other industries are gaining advanced benefits from them are all drivers for increased utilisation [20]. For these reasons, it is essential to recognise the challenges and risks associated with the use of technologies to be enable an approach to utilise them in the best way possible.

## Abbreviations

AI: Artificial Intelligence
GDPR: General Data Protection Regulation
HIPAA: Health Insurance Portability and Accountability Act
IoT: Internet of Things
NHS: National Health Service
